# Anticancer properties of propofol-docosahexaenoate and propofol-eicosapentaenoate on breast cancer cells

**DOI:** 10.1186/bcr1036

**Published:** 2005-06-07

**Authors:** Rafat A Siddiqui, Mustapha Zerouga, Min Wu, Alicia Castillo, Kevin Harvey, Gary P Zaloga, William Stillwell

**Affiliations:** 1 Methodist Research Institute, Clarian Health Partners, Indianapolis, IN, USA; 2 Department of Medicine, Indiana University School of Medicine, Indianapolis, IN, USA; 3 Department of Biology, Indiana University-Purdue University, Indianapolis, IN, USA

## Abstract

**Introduction:**

Epidemiological evidence strongly links fish oil, which is rich in docosahexaenoic acid (DHA) and eicosapentaenoic acid (EPA), with low incidences of several types of cancer. The inhibitory effects of omega-3 polyunsaturated fatty acids on cancer development and progression are supported by studies with cultured cells and animal models. Propofol (2,6-diisopropylphenol) is the most extensively used general anesthetic–sedative agent employed today and is nontoxic to humans at high levels (50 μg/ml). Clinically relevant concentrations of propofol (3 to 8 μg/ml; 20 to 50 μM) have also been reported to have anticancer activities. The present study describes the synthesis, purification, characterization and evaluation of two novel anticancer conjugates, propofol-docosahexaenoate (propofol-DHA) and propofol-eicosapentaenoate (propofol-EPA).

**Methods:**

The conjugates linking an omega-3 fatty acid, either DHA or EPA, with propofol were synthesized and tested for their effects on migration, adhesion and apoptosis on MDA-MB-231 breast cancer cells.

**Results:**

At low concentrations (25 μM), DHA, EPA or propofol alone or in combination had minimal effect on cell adhesion to vitronectin, cell migration against serum and the induction of apoptosis (only 5 to 15% of the cells became apoptotic). In contrast, the propofol-DHA or propofol-EPA conjugates significantly inhibited cell adhesion (15 to 30%) and migration (about 50%) and induced apoptosis (about 40%) in breast cancer cells.

**Conclusion:**

These results suggest that the novel propofol-DHA and propofol-EPA conjugates reported here may be useful for the treatment of breast cancer.

## Introduction

Omega-3 polyunsaturated long-chain fatty acids (ω-3 PUFAs) have been documented to inhibit or even prevent cancer. Epidemiological evidence strongly links fish oil (rich in docosahexaenoic acid (DHA) and eicosapentaenoic acid (EPA)) with low incidences of several types of cancer [1-6]. The inhibitory effects of ω-3 PUFAs on cancer development and progression are supported by studies using cultured cells and animal models [7-14]. However, the mechanisms by which ω-3 PUFAs inhibit cancer remain unclear. Of particular interest are the many reports demonstrating anticancer properties of ω-3 PUFAs on the growth and survival of various cancer cell lines cultured *in vitro*. Included in the growing list of affected cell lines are breast cancer cells [7,8,15-17]. Several of these reports indicate that at low concentrations ω-3 PUFAs (10 to 100 μM) produce anticancer effects through the induction of apoptosis rather than via cytotoxicity. Propofol (2,6 diisopropylphenol) is the most extensively used general anesthetic-sedative agent employed today [18,19] and is nontoxic to humans at high levels (3 to 8 μg/ml; 20 to 50 μM) [20]. Propofol is a potent antioxidant [21-24] and has been shown to stimulate protein kinase C [25,26], inhibit calcium entry in muscle cells [27] and increase the calcium sensitivity of myofilaments in ventricular myocytes [28]. Propofol is also a potent direct vasodilator and bronchodilator and has recently been shown to possess anti-inflammatory and antiseizure properties. Although the exact signaling systems responsible for these effects are unclear, it does indicate that propofol alters signaling pathways within cells.

Although most studies concerning the mode of action of propofol have concentrated on its action as an anesthetic, there are a few reports indicating that this compound may also affect cellular processes related to cancer. Clinically relevant concentrations of propofol (3 to 8 μg/ml) were reported to decrease the metastatic potential of human cancer cells, including HeLa, HT1080, HOS and RPMI-7951 cells [29]. In addition, continuous infusion of propofol inhibited pulmonary metastasis of murine osteosarcoma (LM8) cells in mice through the modulation of Rho A [29]. In HL-60 human promyelocytic leukemia cells, propofol was shown to inhibit growth and induce the formation of apoptotic bodies, increase DNA fragmentation and laddering, activate caspase-3, caspase-6, caspase-8 and caspase-9, and induce the cytosolic release of cytochrome *c *[30]. The conclusion from these studies was that propofol induces apoptosis through both a cell-surface death receptor (extrinsic) and the mitochondrial (intrinsic) pathway. These studies suggest that propofol possesses anticancer properties in addition to its sedative effects.

The omega-3 PUFAs DHA and EPA are natural nontoxic food substances that have interesting anticancer properties. Propofol is a widely employed nontoxic anesthetic that also has anticancer properties. Although several preparations of propofol with lipid mixtures are available for anesthetic usage (AstraZeneca, Wilmington, DE), to our knowledge propofol has not previously been covalently conjugated with fatty acids. The purpose of this study was to synthesize and investigate novel compounds composed of DHA or EPA conjugated to propofol. The conjugates were tested for their ability to inhibit breast cancer cell migration, alter adhesion to the matrix protein vitronectin and induce apoptosis. The results indicate that these novel conjugates might represent a new class of anticancer agents.

## Materials and methods

### Materials

MDA-MB-231 breast cancer cells were purchased from the American Type Culture Collection (ATCC; Manassas, VA). The Vybrant Apoptosis assay kit was from Molecular Probes (Eugene, OR), and DMEM, penicillin, streptomycin and glutamine were from Invitrogen Corporation (Grand Island, NY). Fetal bovine serum was from BioWhittaker (Walkersville, MD). Transwell chemotaxis chamber plates were from Corning Incorporated (Corning, NY). Cytomatrix Human Vitronectin-Coated Strips were from Chemicon International, Inc. (Temecula, CA). DHA and EPA fatty acid standards for thin-layer chromatography (TLC) and gas chromatography (GC) were from Nu-Chek Prep, Inc. (Elysian, MN). Methanol, chloroform, petroleum ether, diethyl ether, acetic acid, hexane and ethanol were from Fisher Scientific (Fair Lane, NY). Propofol, *N*,*N*-dicyclohexylcarbodiimide, 2,6-di-tert-butyl-4-methylphenol (BHT), 4-(dimethyl amino)pyridine, hematoxylin, crystal violet and all other reagents were from Sigma Chemical Co. (St Louis, MO).

### Synthesis and purification of propofol-DHA and propofol-EPA

Although only propofol-DHA synthesis is described here, the procedure is analogous for the propofol-EPA conjugate. Synthesis was performed in two steps. First, docosahexaenoic acid anhydride (DHA-anhydride) was synthesized, followed by its esterification to propofol. Synthesis of the conjugate was performed under reduced light and under nitrogen to minimize auto-oxidation. In brief, DHA or EPA (0.300 mmol), a coupling reagent, *N*,*N*-dicyclohexylcarbodiimide (0.45 mmol), and an antioxidant, BHT (1.5 μM), were dissolved in 5 ml of chloroform and the reaction was stirred for 60 min at room temperature (23–25°C). To this mixture, propofol (0.29 mmol) and 4-(dimethyl amino)pyridine (0.152 mmol) were added. The mixture was stirred for a period of 12 hours; the suspension was then filtered, washed with light petroleum (38.3–53.2°C) and subjected to purification on analytical thin-layer plates (silica gel, 60 A, 0.2 mm thickness; Alltech Associates, Inc., Deerfield, IL). The plates were developed in a solvent mixture of petroleum ether and ethyl acetate (92:8, v/v) and the products were revealed with iodine vapor. The spots were compared with authentic DHA, propofol, BHT, pyridine and *N*,*N*-dicyclohexylcarbodiimide and the spot corresponding to the new compound was scraped, suspended in chloroform/methanol (20:80, v/v), passed through a glass filter and stored at -70°C. The propofol-DHA and propofol-EPA conjugates were characterized as described below.

### Characterization of propofol-DHA

Characterization of the propofol-DHA conjugate was performed by a combination of techniques. First, the presence of propofol in the conjugate was assessed by UV spectroscopy with an SLM Aminco 3000 spectrophotometer. The conjugate was dissolved in ethanol and the absorption spectra was measured from 200 to 600 nm. Next, the new compound was hydrolyzed and methylated in 3 M methanolic HCl [31]. The product of the reaction was extracted with hexane/water (2:1, v/v) and the organic phase was analyzed with a Shimatzu 17A gas chromatograph with a 0.25 mm × 30 m Stabilwax capillary column (Resteck, Belfont, PA). The temperature ramp was 180 to 240°C at 3°C/min (hold 3 min), followed by 240 to 245°C at 1°C/min. To determine the presence of a possible ester group formed in the new product, an infrared spectrum was taken on a Perkin Elmer/2000FT-IR spectrometer. A thin film of the product was made by evaporation from chloroform. Finally, the molecular mass of the product was determined by mass spectrometry on a MAT95XP mass spectrometer (Thermo Electron Corp., San Jose, CA) by Dr Jonathan Karty at the mass spectrometry facility of Indiana University (Bloomington, IN) using an electrospray method.

### Cell culture

MDA-MB-231 breast cancer cells were grown in DMEM containing 10% fetal bovine serum, 100 units/ml penicillin and 100 μg/ml streptomycin at a density of 10^6 ^cells/ml for routine culture. For experimental purposes, cells were cultured at the cell density indicated and treated with DHA, EPA, propofol or the conjugates. The test compounds were stored in hexane at -80°C. An aliquot of the conjugate was dried under nitrogen and the compounds were diluted in ethanol just before use. The final concentration of ethanol (less than 0.1%) in the treated cultures did not exhibit any cytotoxic effects as measured by lactate dehydrogenase release and a WST-cell proliferation assay (results not shown).

### Cell growth assay

The effect of the fatty acids on cell growth was determined with a WST-1 assay in accordance with the manufacturer's instructions (Roche Biosciences, Indianapolis, IN).

### Cell migration assay

Cell migration was performed with Transwell Chemotaxis Chamber Plates (Corning Inc.). The bottom chamber was supplemented with 300 μl of DMEM containing 10% fetal calf serum, and 100 μl of MDA-MB-231 cells (10^5^/ml) in serum-free DMEM was added to the top chamber with or without (control) test compounds. The plates were incubated for 4 hours at 37°C in a humidified CO_2 _incubator. After incubation, the insides of the inserts were washed and cleaned with cotton swabs, and the filters were fixed with 5% formaldehyde. The cells were stained with hematoxylin as described [32] and cells that migrated through the filters were counted under a microscope on the bottom side of the filters.

### Cell adhesion assay

The cell adhesion assay was performed with Cytomatrix human vitronectin-coated strips (Chemicon International, Inc., Temecula, CA). The strips were incubated with 100 μl of MDA-MB-231 cells (10^5^/ml) at 37°C for 45 min in a CO_2 _incubator under serum-free conditions with control or test compounds. The wells were washed three times with PBS and the adhered cells were stained with 0.2% crystal violet in 10% ethanol for 5 min at room temperature. The excess stain was removed by washing six times with PBS. The stained cells were dissolved in 100 μl of solubilization buffer (1:1 mixture of 0.1 M NaH_2_PO_4_, pH 4.5, and 50% ethanol) and the absorbance was read at 540 nm. Absorbance of dye in the control (vehicle-treated) cells was regarded as 100% adherence and the percentage adherence of treated cells was calculated in comparison with that of the control cells.

### Cell viability and apoptosis assay

A Vybrant apoptosis assay kit (Molecular Probes) was used in accordance with the manufacturer's protocol. In brief, 100 μl of MDA-MB-231 cells (10^5^/ml) suspended in serum-free DMEM were incubated in 96-well plates at 37°C in a humidified CO_2 _incubator with control or test compounds. At the end of the incubation period (24 hours), reagents A (YO-PRO-1, 100 μM) and B (propidium iodide, 1 mg/ml) from the kit were added to each well (1 μl/ml) and the plates were left to incubate for 30 min on ice. The cells were revealed by using a fluorescence microscope with appropriate filters. Live cells do not exhibit any fluorescence because the dyes are impermeable to living cells, dead and necrotic cells exhibit red fluorescence, and apoptotic cells fluoresce green. Total and apoptotic cells were counted and the percentage of cells exhibiting apoptosis was calculated.

### Caspase-3 activation

Activation of caspase-3 in MDA-MB-231 cells was determined with a caspase-3 activity assay kit (Oncogenes Research Products, San Diego, CA). Cells (5 × 10^5 ^per well) were grown in 48-well plates in serum-free DMEM for 24 hours at 37°C in a humidified CO_2 _incubator with control or test compounds. After incubation, a caspase-3 fluorescent substrate (Asp-Glu-Val-Asp-fluoromethylketone-aminotrifluoromethylcoumarin conjugate (DEVD-AFC)) was added to each well (10 μl per well) and the plates were incubated for a further 1 hour. Cells were revealed under a fluorescence microscope and pictures were taken with a MagnaFire charge-coupled device camera (Optronics, Goleta, CA).

### Cytochrome *c *release assay

Induction of apoptosis was also assayed by detecting cytochrome *c *release from the apoptotic cells by western blot analysis as described [33]. Cells (5 × 10^5 ^per well) were grown in 48-well plates in serum-free DMEM for 24 hours at 37°C in a humidified CO_2 _incubator with control or test compounds. Cells were then homogenized in a buffer containing 10 mM HEPES, pH 7.4, 0.25 mM sucrose and 1 mM EDTA; a post-nuclear fraction was prepared by centrifugation at 2,000 *g *for 5 min at 4°C. The supernatant was further centrifuged at 100,000 *g *for 20 min at 4°C and the resultant cytosolic fraction was used for cytochrome *c *detection by immunoblotting. Proteins were separated by 8 to 15% SDS-PAGE and the blot was incubated with monoclonal anti-cytochrome *c *(BD Bioscience Pharmingen, San Diego, CA) or monoclonal antibodies against glyceraldehyde-3-phosphate dehydrogenase (1:1,000 dilution; Santa Cruz Biotech, Santa Cruz, CA) overnight at 4°C and detected with secondary anti-mouse peroxidase-conjugated antibodies (Amersham Pharmacia Biotech, Little Chalfont, Buckinghamshire, UK). The bands were detected with a chemiluminescence detection kit (Pierce, Rockford, IL). The relative distributions of cytochrome *c *and glyceraldehyde-3-phosphate dehydrogenase (loading control) were determined by densitometric analysis with the Kodak imaging system (Eastman Kodak Company, Rochester, NY).

### Statistical analysis of data

For each experiment, means and standard errors were found for each treatment group and were plotted accordingly. Analysis of variance was performed to test for an overall effect across treatments. Individual treatments were tested against the control by using Dunnett's multiple comparison test to control the Type I experimental wise error. Analyses were conducted with SAS version 8.2 (SAS Institute, Cary, NC).

## Results

### Characterization of propofol-docosahexaenoate

**Figure 1:**
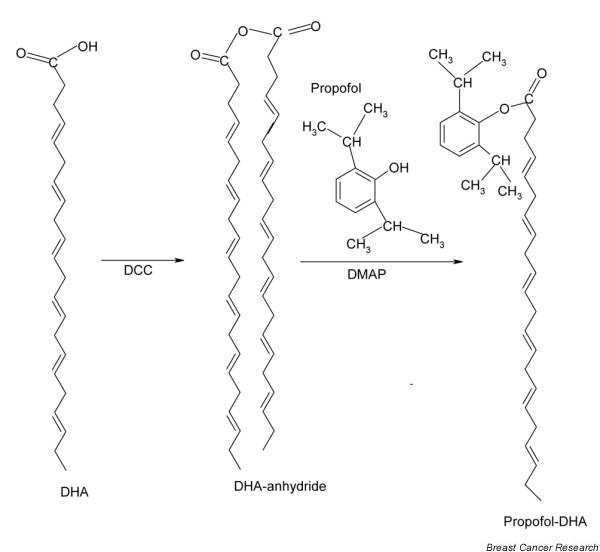
Chemical synthesis of the propofol-docosahexaenoic acid (propofol-DHA) conjugate. Synthesis was performed in two steps. First, docosahexaenoic acid anhydride (DHA-anhydride) was synthesized with a coupling reagent, *N*,*N*-dicyclohexylcarbodiimide (DCC); subsequently, conjugation of propofol with DHA was performed in the presence of 4-(dimethylamino)pyridine (DMAP).

**Figure 2:**
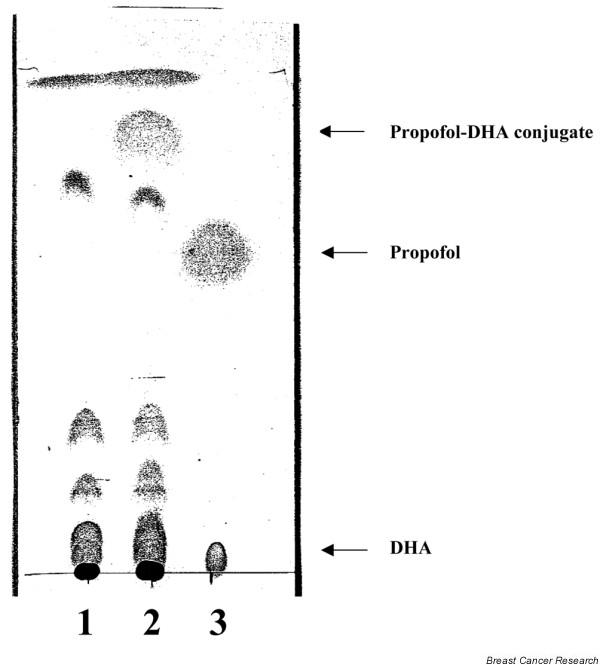
Thin-layer chromatography (TLC) analysis of propofol-docosahexaenoic acid (propofol-DHA) conjugate synthesis. Synthesis of propofol-DHA was performed as described in the text. The product of the reaction was separated by TLC with a light petroleum (38.3–53.2°C)/ethyl ether (98:2, v/v) solvent system and the products were revealed with iodine vapors. Lane 1, product of the reaction mixture that contained all starting material except propofol; lane 2, product of the complete reaction mixture; lane 3, propofol and DHA standards.

The chemical synthesis of the propofol-DHA conjugate is shown in Fig. 1. The initial isolation and characterization of the synthetic propofol-DHA conjugate were performed with TLC. The results shown in Fig. 2 demonstrate that reaction between DHA and propofol resulted in the formation of a new product with an *R*_*F *_of 0.90. The *R*_*F *_of this product is very different from that of either DHA or propofol. The band corresponding to the new compound was scraped and subjected to spectrometric characterization. The propofol spectra showed two absorption peaks, at 216 and 274 nm; these peaks were shifted to 205 and 265 nm, respectively, for the propofol-DHA conjugate (data not shown). Next, the conjugate was hydrolyzed and then methylated for analysis by gas chromatography. The results demonstrate that the commercially available propofol (Aldrich-Sigma Chemical Co.) was 97% pure, and analysis of the hydrolyzed product of the propofol-DHA conjugate showed a DHA : propofol ratio of 1: 0.8. The ratio varied from the expected 1:1 because other isoforms of propofol were also conjugated to DHA (data not shown).

**Figure 3:**
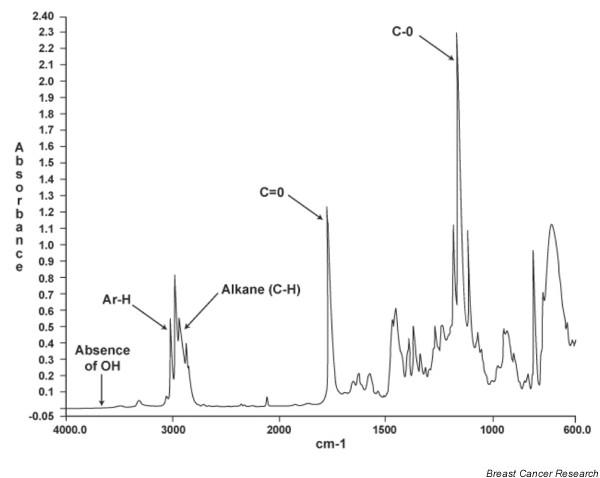
Characterization of propofol-docosahexaenoic acid conjugate by infrared spectroscopy. The infrared spectrum of the conjugate was recorded on a Perkin Elmer/2000 FT-IR. The sample was run as a thin film after evaporation of solvent. The broad, strong absorption bands at 1,750 cm^-1 ^and 1,250 cm^-1 ^are attributable to C=O and C–O bonds, respectively, and indicate the presence of an ester. The band at 3,030 cm^-1 ^is characteristic of an aromatic C–H (propofol) and the band at 2,800 to 2,960 cm^-1 ^is characteristic of aliphatic C–H bonds. No O–H absorption band was seen, indicating the absence of nonesterified propofol.

Further characterization of the conjugate was performed by infrared spectroscopy. The infrared absorption spectra of the propofol-DHA conjugate (Fig. 3) showed two broad, strong absorption bands at 1,750 and 1,250 cm^-1^, which are attributable to C = O and C–O bonds, respectively, indicating the presence of an ester. The band at 3,030 cm^-1 ^is characteristic of an aromatic C–H bond (propofol), and the band at 2,800 to 2,960 cm^-1 ^is characteristic of aliphatic C–H bonds. No O–H absorption band was seen, indicating the absence of non-esterified propofol. The presence of an ester bond, aromatic C–H absorbance, and absence of free O–H group absorbance further confirms the formation of a propofol-DHA conjugate.

The propofol-EPA conjugate was also synthesized; its characterization by TLC, UV spectroscopy, GC analysis of hydrolyzed product and infrared spectroscopy was performed in a similar fashion (data not shown).

Finally, a mass spectroscopic analysis was performed to determine the molecular mass of the product on a MAT 95XP high-resolution, high-mass-accuracy mass spectrometer. Its high mass accuracy (less than 5 p.p.m. in magnetic scan and less than 2 p.p.m. in electric scan) enables its data to be used to provide a formula match. That is, the mass spectra are precise enough to verify a chemical formula based on the sum of the mass defects from the constituent atoms. Results demonstrate that the propofol-DHA mass spectra identified a product with a parent molecular mass of 488.36 Da, which is very close to the calculated molecular mass, 488.74 Da. The observed monoisotopic molecular mass was within 0.8 part per million (0.0004 atomic mass units) of that predicted for propofol-DHA. The propofol-EPA spectra identified a product of molecular mass 462.34 Da, which is very close to the calculated molecular mass of 462.70 Da. The observed monoisotopic molecular mass was within 1.1 parts per million (0.0006 atomic mass units) of that predicted for propofol-EPA (see mass spectrometry data as additional file 1).

Characterization of the propofol-DHA conjugate and propofol-EPA conjugates suggests that the coupling reaction between DHA or EPA and propofol resulted in the formation of new products. The conjugates are a one-to-one ester of DHA or EPA and propofol.

### Effect of DHA, EPA and propofol on cell growth

**Figure 4:**
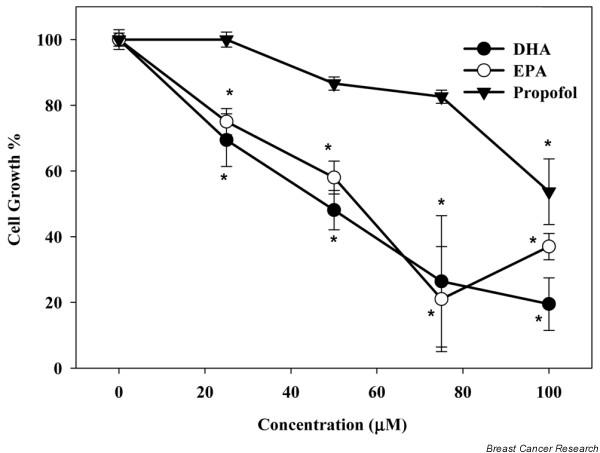
Effect of docosahexaenoic acid (DHA), eicosapentaenoic acid (EPA) and propofol on cellular growth. Cells (10^4 ^per well) were seeded overnight in a 96-well plate and then treated for 24 hours with various concentrations of DHA, EPA or propofol in serum-free medium. Cell growth was assayed with a WST-1 assay as described in the Materials and methods section. Results are means ± SEM for three experiments. The results were analyzed by analysis of variance and Dunnett's multiple comparison test to control the Type I experimental wise error. Significant differences from the control (*P *< 0.05) are indicated with an asterisk.

Results shown in Fig. 4 demonstrate that at 25 μM DHA or EPA significantly inhibited MDA-MB-231 breast cancer cell growth by 20 to 30%, whereas only at high concentration (100 μM) did propofol significantly inhibit breast cancer cell growth. Subsequent experiments were therefore performed with a concentration of 25 μM for DHA, EPA, propofol, propofol-DHA and propofol-EPA.

### Effect of the conjugates on breast cancer cell migration

**Figure 5:**
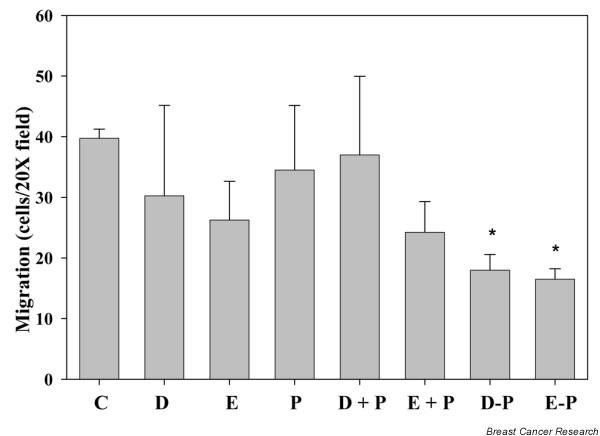
Effect of propofol-docosahexaenoic acid (propofol-DHA) and propofol-eicosapentaenoic acid (propofol-EPA) conjugates on cell migration. MDA-MB-231 cells (10^4^) were incubated for 4 hours with 25 μM DHA (D), 25 μM EPA (E), 25 μM propofol (P), 25 μM each of D+P, 25 μM each of E+P, and 25 μM propofol-DHA (D-P) or 25 μM propofol-EPA (E-P) using transwell plates. The control cells (C) were treated with equal amounts of ethanol. Cells that migrated through the filter were counted under a microscope as described in the text. Results are means ± SEM for four experiments. The results were analyzed by analysis of variance and Dunnett's multiple comparison test to control the Type I experimental wise error. Significant differences from the control (*P *< 0.05) are indicated with an asterisk.

Because MDA-MB-231 breast cancer cells are highly invasive, we monitored the effect of the conjugates on cell migration. The results shown in Fig. 5 demonstrate that DHA, EPA and propofol alone or in combination do not have a significant effect on breast cancer cell migration; however, propofol-DHA and propofol-EPA are equally effective and inhibited cell migration by about 50% (*P *< 0.05).

### Effect of the conjugates on breast cancer cell adhesion

**Figure 6:**
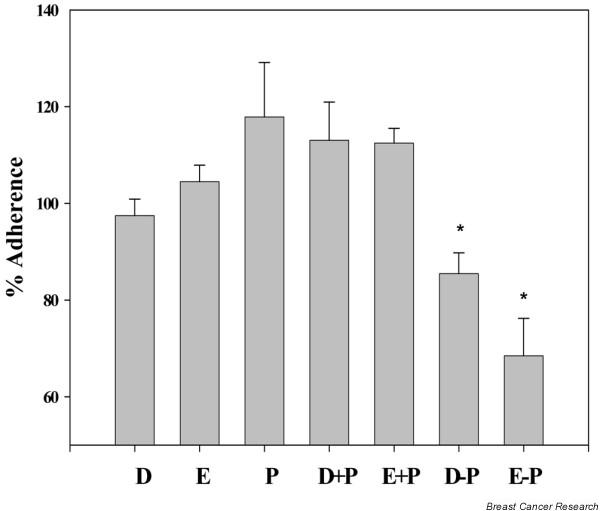
Effect of propofol-docosahexaenoic acid (propofol-DHA) and propofol-eicosapentaenoic acid (propofol-EPA) conjugates on cell adhesion. MDA-MB-231 cells (10^4^) were incubated in vitronectin-coated plates in the presence of 25 μM DHA (D), 25 μM EPA (E), 25 μM propofol (P), 25 μM each of D+P, 25 μM each of E+P, and 25 μM propofol-DHA (D-P) or 25 μM propofol-EPA (E-P) for 45 min. The control cells were treated with equal amounts of ethanol. Cells adhering to the plates were quantified by staining and measurement of the absorbance in a spectrophotometer as described in the text. Results are means ± SEM for four experiments and are presented as the percentages of adhered cells in comparison with the controls. The results were analyzed by analysis of variance and Dunnett's multiple comparison test to control the Type I experimental wise error. Significant differences from the control (*P *< 0.05) are indicated with an asterisk.

We also tested the effect of the propofol-DHA and propofol-EPA conjugates on adhesion of the breast cancer cells to a vitronectin substrate. DHA and EPA alone did not significantly affect breast cancer cell adhesion, whereas propofol itself or in combination with DHA or EPA slightly increased cell adhesion (Fig. 6). In contrast, propofol-DHA and propofol-EPA significantly inhibited cell adhesion by 15% (*P *< 0.05) and 30% (*P *< 0.05), respectively.

### Effect of the conjugates on breast cancer cell apoptosis

**Figure 7:**
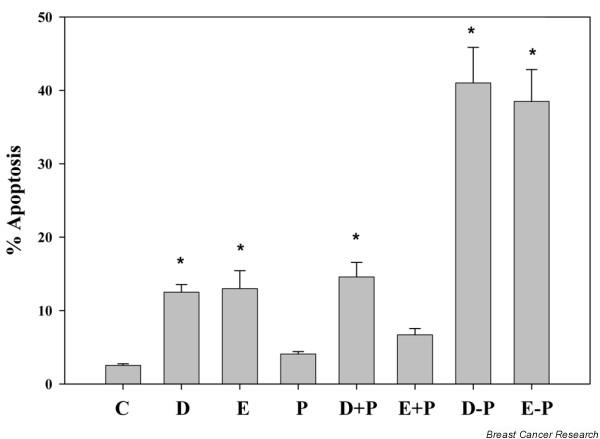
Effect of propofol-docosahexaenoic acid (propofol-DHA) and propofol-eicosapentaenoic acid (propofol-EPA) conjugates on breast cancer cell apoptosis. MDA-MB-231 cells (10^4^) were incubated with 25 μM DHA (D), 25 μM EPA (E), 25 μM propofol (P), 25 μM each of D+P, 25 μM each of E+P, 25 μM propofol-DHA (D-P) or 25 μM propofol-EPA (E-P) for 24 hours. The control cells (C) were treated with equal amounts of ethanol. The apoptotic cells were stained by using the Vybrant Apoptosis assay kit as described in the text. Results are means ± SEM for four experiments. The results were analyzed by analysis of variance and Dunnett's multiple comparison test to control the Type I experimental wise error. Significant differences from the control (*P *< 0.05) are indicated with an asterisk.

**Figure 8:**
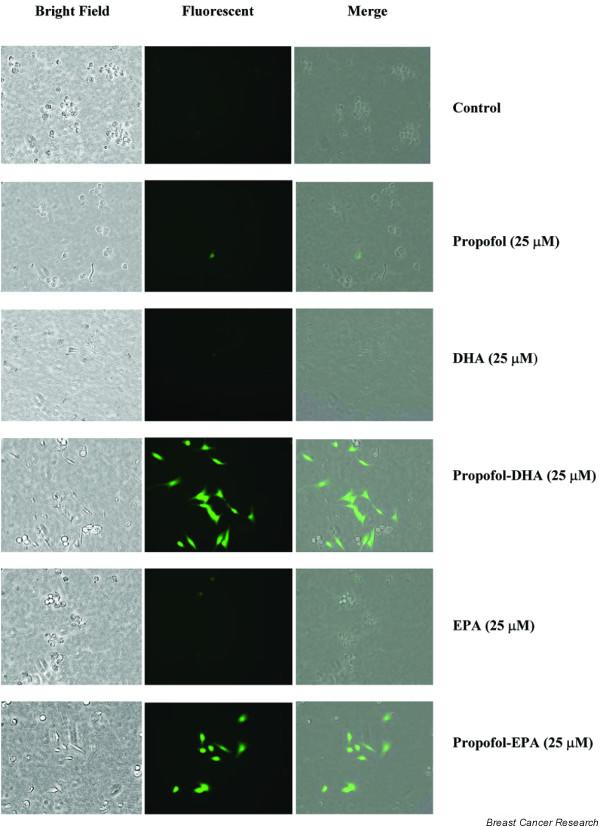
Effect of propofol-docosahexaenoic acid (propofol-DHA) and propofol-eicosapentaenoic acid (propofol-EPA) conjugates on caspase-3 activation. MDA-MB-231 cells (5 × 10^5 ^per well) were grown in 96-well plates for 24 hours at 37°C in a humidified CO_2 _incubator with control or test compounds. Cells containing activated caspase-3 fluoresced green as the result of cleavage of a fluorogenic substrate (Asp-Glu-Val-Asp-fluoromethylketone-aminotrifluoromethylcoumarin conjugate).

Propofol-DHA and propofol-EPA were tested for their ability to initiate apoptosis within MDA-MB-231 breast cancer cells. Induction of apoptosis was assayed by incubating the cells with DHA, EPA or propofol alone or mixtures of DHA and EPA with propofol as well as with the propofol-DHA and propofol-EPA conjugates. Results shown in Fig. 7 indicate that, at 25 μM, DHA, EPA or propofol alone induced apoptosis in only 5 to 15% of the cells. Incubating DHA or EPA with propofol did not further enhance apoptosis, whereas propofol-DHA or propofol-EPA conjugates were strongly apoptotic, inducing apoptosis in about 40% (*P *< 0.05) of the breast cancer cells. To confirm the apoptotic response in these cells, we further assayed for caspase-3 activity, a protease involved in the execution phase of apoptosis. The assay was performed by using a specific fluorescent caspase-3 substrate (DEVD-AFC) that on hydrolysis produces a green fluorescent product. The results shown in Fig. 8 demonstrate that cells incubated with DHA, EPA or propofol have only a few caspase-3 positive cells (2 to 5%), whereas many more cells treated with propofol-DHA or propofol-EPA conjugates exhibited caspase-3 activation (15 to 20%).

**Figure 9:**
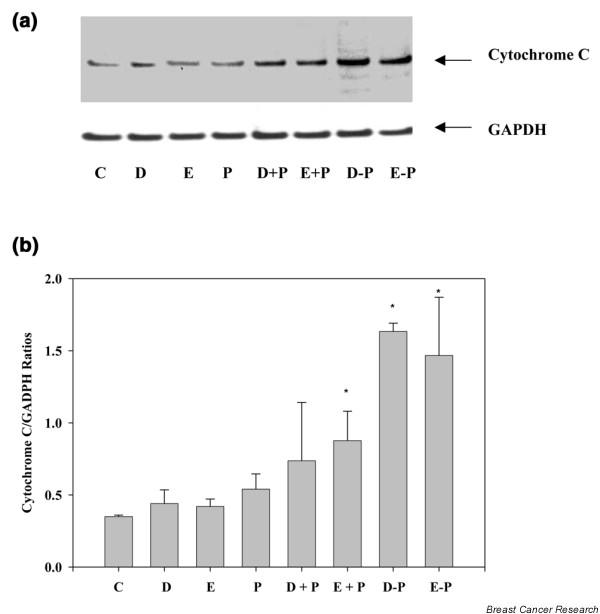
Effect of propofol-docosahexaenoic acid (propofol-DHA) and propofol-eicosapentaenoic (propofol-EPA) conjugates on cytochrome *c *release. MDA-MB-231 cells (0.5 × 10^6^/well) were grown in 96-well plates for 24 hours at 37°C in a humidified CO_2 _incubator with control or test compounds. **(a) **Postnuclear supernatant was used for the determination of cytochrome *c *release using immunobloting as described in the Materials and Methods section. The relative distributions of cytochrome *c *and glyceraldehyde-3-phosphate dehydrogenase (loading control) were determined. **(b) **Quantification was by densitometry analysis with the Kodak imaging system. Results are means ± SEM for three experiments. The results were analyzed by analysis of variance and Dunnett's multiple comparison test to control the Type I experimental wise error. Significant differences from the control (*P *< 0.05) are indicated with an asterisk. D, 25 μM DHA; E, 25 μM EPA; P, 25 μM propofol; D+P, 25 μM DHA plus 25 μM propofol; E+P, 25 μM EPA plus 25 μM propofol; D-P, 25 μM propofol-DHA; E-P, 25 μM propofol-EPA.

The effect of propofol-DHA and propofol-EPA conjugates on apoptosis was further analyzed by assaying for cytochrome *c *release (Fig. 9). DHA, EPA and propofol alone had no significant effect on cytochrome *c *release, whereas DHA or EPA with propofol caused an approximately 150 to 200% (*P *< 0.05) increase in cytochrome *c *release. However, propofol-DHA or propofol-EPA conjugates caused significant increases in cytochrome *c *release by about 300 to 400% (*P *< 0.05).

These results confirm that the propofol-DHA and propofol-EPA conjugates are far more effective at inducing apoptosis in breast cancer cells than are the unconjugated parent compounds DHA, EPA or propofol.

## Discussion

Often a major obstacle to the successful use of a drug is its ability to be taken up and retained by cells. Either the drug must have its target on the outer membrane surface or it must cross the plasma membrane through either an existing transport system or by simple diffusion to affect intracellular targets. One approach to overcoming the problem of cell entry and retention has been to link water-soluble drugs to lipophilic carriers. Several attempts have been made in the past to synthesize novel compounds by conjugating fatty acids with drugs. For example, chlorambucil-fatty acid conjugates (Chl-fatty acid) were synthesized and tested on human lymphoma cell lines [34]. These studies found that the conjugates (including those with DHA) selectively affected neoplastic lymphocytes, with minimal effect on quiescent lymphocytes [34]. The cell toxicity observed with Chl-arachidonic acid and Chl-docosahexaenoic acid against lymphoma cells was equal to or higher than the individual toxic potential of either chlorambucil or the fatty acids, whereas the Chl-oleic acid conjugate was much less toxic than Chl alone. The authors concluded that the coupling of chlorambucil with polyunsaturated fatty acids was selective against neoplastic versus quiescent lymphocytes [34]. During the present investigation, propofol was conjugated with the omega-3 fatty acids DHA and EPA. It is possible that propofol conjugated with arachidonic acid, an omega-6 polyunsaturated fatty acid, or with a saturated fatty acid might be as effective. However, there is now a large body of evidence that DHA and EPA are far more effective than shorter and less unsaturated fatty acids [35]. Clearly additional studies are needed to establish the specificity of propofol conjugates with different fatty acids.

Similarly, DHA-paclitaxel was synthesized by Bradley and colleagues, who demonstrated that the conjugate possessed antitumor activity in mice with lung tumors [36]. In the M109 mouse tumor model, DHA-paclitaxel was less toxic to animals than paclitaxel alone and cured all tumor-bearing animals; in contrast, unconjugated paclitaxel cured none. This study indicated a limited plasma area under the drug concentration–time curve (AUC) for paclitaxel, and an increase in tumor AUC of DHA-paclitaxel administration was consistent with the increase in therapeutic index of DHA-paclitaxel relative to paclitaxel in the M109 mouse tumor model. During the present investigation we did not measure the tissue concentrations of propofol, DHA or EPA after treatment with propofol-DHA or propofol-EPA, but we plan to do so in a further study.

We have previously employed the DHA-conjugate approach to enhance the availability and hence the activity of the anticancer drug methotrexate [37]. Two phosphatidylcholines were synthesized to contain methotrexate in the *sn*-2 position and either stearic acid or DHA in the *sn*-1 position. The DHA-containing and methotrexate-containing phosphatidylcholines were more effective than conjugates containing stearic acid. Synthesis of phosphatidylcholines containing DHA and propofol is also a possibility, but such a synthesis is more cumbersome. In the study described here, we therefore used another approach to synthesize a class of novel compounds by directly conjugating DHA or EPA with propofol. Results presented in Figs 1 to 4 show that our synthetic process produced the propofol-DHA and propofol-EPA conjugates. Separations by TLC (Fig. 2), UV absorption, hydrolysis and GC analysis (not shown), infrared spectroscopy (Fig. 3) and mass spectroscopy (not shown) confirm the identity of the conjugates.

After obtaining the conjugates, we investigated their anticancer effects on breast cancer cells. MDA-MB-231 is a highly invasive breast cancer cell line that has the potential for uncontrolled proliferation and metastasis in animal models [38]. We investigated the effects of these novel conjugates on cell migration, adhesion and cancer cell death through apoptosis. Results presented in Fig. 5 show that the conjugates do indeed inhibit cell migration, whereas DHA, EPA or propofol at the same concentration has no effect. We then assayed these compounds for their effect on cell adhesion to a vitronectin substrate. Results in Fig. 6 show that DHA or EPA alone or in combination with propofol did not inhibit cancer cell adhesion, whereas the same concentrations of propofol-DHA and propofol-EPA caused significant inhibition. Cell migration and adhesion are essential processes in tumor metastasis [39]. The results of this study suggest that these novel conjugates are able to affect the metastatic potential of this breast cancer cell line by inhibiting both migration and adhesion at concentrations at which DHA, EPA or propofol alone are not effective.

We then further analyzed the effect on these conjugates on the induction of apoptosis. Results in Fig. 7 show that both conjugates are able to induce apoptosis (Vybrant apoptosis assay kit) in breast cancer cells. Induction of apoptosis was further confirmed by assaying for caspase-3 activation (Fig. 8) and cytochrome *c *release (Fig. 9). Cytochrome *c *has a function in the intrinsic pathway of apoptosis and leads to the activation of caspase-3, which is a downstream enzyme in the apoptosis process and is involved in the execution phase of the death pathway [40]. Release of cytochrome *c *and activation of caspase-3 by propofol-DHA and propofol-EPA conjugates confirm that these compounds induce a cell signaling pathway for apoptosis that eventually leads to the death of breast cancer cells.

During the present study, the detailed molecular mechanisms by which these conjugates inhibit migration and adhesion and induce apoptosis were not investigated. Our previous studies [41-43] and other reports [44,45] have shown that DHA is rapidly taken up by cells and incorporated into membrane phospholipids. Propofol, being a partly lipophilic agent, also interacts with cellular membranes [46]. However, because of its volatile nature, it is rapidly removed from membranes and has a very short half-life in the circulation [19]. It is possible that these conjugates provide a mechanism whereby propofol can be retained in cell membranes for a longer duration and therefore enhance its anticancer effects. Studies have shown that the incorporation of DHA into membranes results in reorganization and the formation of membrane microdomains [47]. These conjugates might therefore influence cell-signaling pathways involved in cell migration, adhesion and apoptosis.

We have previously shown that DHA induces apoptosis in Jurkat leukemic cells by activating protein phosphatases [48,49]. We have not yet tested this possibility with the conjugates; however, it is likely that these conjugates also activate protein phosphatases, inducing apoptosis. Further investigation is required to explore the molecular mechanisms by which propofol-DHA and propofol-EPA conjugates affect the growth, migration and adhesion of breast cancer cells. We also plan to investigate the effect of the conjugates on other cancer cell lines. Importantly, our preliminary observations indicate that similar concentrations of these conjugates do not induce any cytotoxic effects in normal skeletal muscle cells, cardiomyocytes or hepatocyte cell lines from rat (data not shown). Further studies are required to test these compounds on normal human cell lines.

## Conclusion

Our synthesis has yielded two novel lipid compounds, namely propofol-DHA and propofol-EPA. These conjugates exhibit anticancer effects that include the inhibition of cell migration and adhesion and the induction of apoptosis within MDA-MB-231 breast cancer cells. The conjugates are more active than the parent compounds and possess unique anticancer activities not found with the latter (namely inhibition of adherence). These conjugates were not tested in other breast cancer cell lines or other cancers of different anatomical locations. Experiments are under way to test these conjugates on different cancer cells lines and also in model systems *in vivo*.

## Abbreviations

BHT = 2,6-di-tert-butyl-4-methylphenol; Chl = chlorambucil; DEVD-AFC = Asp-Glu-Val-Asp-fluoromethylketone-aminotrifluoromethylcoumarin conjugate; DHA = docosahexaenoic acid; DMEM = Dulbecco's modified Eagle's medium; EPA = eicosapentaenoic acid; GC = gas chromatography; ω-3 PUFAs = omega-3 polyunsaturated fatty acids; TLC = thin-layer chromatography.

## Competing interests

The author(s) declare that they have no competing interests.

## Authors' contributions

RAS, GZ, and SW designed conjugates, supervised experiments and contributed in manuscript writing. MZ led the propofol-DHA and propofol-EPA synthesis and characterization. MW, AC, and KH were responsible for laboratory work for adhesion, migration and apoptosis assays. All authors read and approved the final manuscript.

## Supplementary Material

Additional File 1 A Word document containing mass spectrometry data for propofol-DHA and propofol-EPA conjugates are presented as additional file 1. Click here for file

## References

[B1] Bang HO, Dyerberg J (1972). Plasma lipids and lipoproteins in Greenlandic west coast Eskimos. Acta Med Scand.

[B2] Vogel VG, McPherson RS (1989). Dietary epidemiology of colon cancer. Hematol Oncol Clin North Am.

[B3] Kaizer L, Boyd NF, Kriukov V, Tritchler D (1989). Fish consumption and breast cancer risk: an ecological study. Nutr Cancer.

[B4] Schloss I, Kidd MS, Tichelaar HY, Young GO, O'Keefe SJ (1997). Dietary factors associated with a low risk of colon cancer in coloured west coast fishermen. S Afr Med J.

[B5] Berg JP, Glattre E, Haldorsen T, Hostmark AT, Bay IG, Johansen AF, Jellum E (1994). Longchain serum fatty acids and risk of thyroid cancer: a population-based case-control study in Norway. Cancer Causes Control.

[B6] Terry P, Lichtenstein P, Feychting M, Ahlbom A, Wolk A (2001). Fatty fish consumption and risk of prostate cancer. Lancet.

[B7] Noguchi M, Minami M, Yagasaki R, Kinoshita K, Earashi M, Kitagawa H, Taniya T, Miyazaki I (1997). Chemoprevention of DMBA-induced mammary carcinogenesis in rats by low-dose EPA and DHA. Br J Cancer.

[B8] Rose DP, Connolly JM, Coleman M (1996). Effect of omega-3 fatty acids on the progression of metastases after the surgical excision of human breast cancer cell solid tumors growing in nude mice. Clin Cancer Res.

[B9] Jenski LJ, Zerouga M, Stillwell W (1995). Omega-3 fatty acid-containing liposomes in cancer therapy. Proc Soc Exp Biol Med.

[B10] Norrish AE, Skeaff CM, Arribas GL, Sharpe SJ, Jackson RT (1999). Prostate cancer risk and consumption of fish oils: a dietary biomarker-based case-control study. Br J Cancer.

[B11] Calviello G, Palozza P, Maggiano N, Franceschelli P, Di Nicuolo F, Marcocci ME, Bartoli GM (1999). Effects of eicosapentaenoic and docosahexaenoic acids dietary supplementation on cell proliferation and apoptosis in rat colonic mucosa. Lipids.

[B12] Calviello G, Palozza P, Maggiano N, Piccioni E, Franceschelli P, Frattucci A, Di Nicuolo F, Bartoli GM (1999). Cell proliferation, differentiation, and apoptosis are modified by *n*-3 polyunsaturated fatty acids in normal colonic mucosa. Lipids.

[B13] Calviello G, Palozza P, Piccioni E, Maggiano N, Frattucci A, Franceschelli P, Bartoli GM (1998). Dietary supplementation with eicosapentaenoic and docosahexaenoic acid inhibits growth of Morris hepatocarcinoma 3924A in rats: effects on proliferation and apoptosis. Int J Cancer.

[B14] Madhavi N, Das UN (1994). Effect of *n*–6 and *n*–3 fatty acids on the survival of vincristine sensitive and resistant human cervical carcinoma cells *in vitro*. Cancer Lett.

[B15] Rose DP, Connolly JM (1999). Antiangiogenicity of docosahexaenoic acid and its role in the suppression of breast cancer cell growth in nude mice. Int J Oncol.

[B16] Hatala MA, Rayburn J, Rose DP (1994). Comparison of linoleic acid and eicosapentaenoic acid incorporation into human breast cancer cells. Lipids.

[B17] Rose DP, Connolly JM (1990). Effects of fatty acids and inhibitors of eicosanoid synthesis on the growth of a human breast cancer cell line in culture. Cancer Res.

[B18] Covington H (1998). Use of propofol for sedation in the ICU. Crit Care Nurse.

[B19] Miller RD, Miller RD (2000). Local anesthetics: anesthesia. Local Anesthetics.

[B20] Coetzee JF, Glen JB, Wium CA, Boshoff L (1995). Pharmacokinetic model selection for target controlled infusions of propofol. Assessment of three parameter sets. Anesthesiology.

[B21] Eriksson O, Pollesello P, Saris NE (1992). Inhibition of lipid peroxidation in isolated rat liver mitochondria by the general anaesthetic propofol. Biochem Pharmacol.

[B22] Murphy PG, Myers DS, Davies MJ, Webster NR, Jones JG (1992). The antioxidant potential of propofol (2,6-diisopropylphenol). Br J Anaesth.

[B23] Tsuchiya M, Asada A, Maeda K, Ueda Y, Sato EF, Shindo M, Inoue M (2001). Propofol versus midazolam regarding their antioxidant activities. Am J Respir Crit Care Med.

[B24] Aarts L, van der Hee R, Dekker I, de Jong J, Langemeijer H, Bast A (1995). The widely used anesthetic agent propofol can replace alpha-tocopherol as an antioxidant. FEBS Lett.

[B25] Hemmings HC, Adamo AI (1994). Effects of halothane and propofol on purified brain protein kinase C activation. Anesthesiology.

[B26] Kanaya N, Gable B, Murray PA, Damron DS (2003). Propofol increases phosphorylation of troponin I and myosin light chain 2 via protein kinase C activation in cardiomyocytes. Anesthesiology.

[B27] Horibe M, Kondo I, Damron DS, Murray PA (2001). Propofol attenuates capacitative calcium entry in pulmonary artery smooth muscle cells. Anesthesiology.

[B28] Kanaya N, Murray PA, Damron DS (2001). Propofol increases myofilament Ca^2+ ^sensitivity and intracellular pH via activation of Na^+^-H^+ ^exchange in rat ventricular myocytes. Anesthesiology.

[B29] Mammoto T, Mukai M, Mammoto A, Yamanaka Y, Hayashi Y, Mashimo T, Kishi Y, Nakamura H (2002). Intravenous anesthetic, propofol inhibits invasion of cancer cells. Cancer Lett.

[B30] Tsuchiya M, Asada A, Arita K, Utsumi T, Yoshida T, Sato EF, Utsumi K, Inoue M (2002). Induction and mechanism of apoptotic cell death by propofol in HL-60 cells. Acta Anaesthesiol Scand.

[B31] Eder K, Reichlmayr-Lais AM, Kirchgessner M (1992). Studies on the methanolysis of small amounts of purified phospholipids for gas chromatographic analysis of fatty acid methyl esters. J Chromatogr.

[B32] Siddiqui RA, English D (2000). Phosphatidylinositol 3'-kinase-mediated calcium mobilization regulates chemotaxis in phosphatidic acid-stimulated human neutrophils. Biochim Biophys Acta.

[B33] Ito A, Uehara T, Tokumitsu A, Okuma Y, Nomura Y (1999). Possible involvement of cytochrome c release and sequential activation of caspases in ceramide-induced apoptosis in SK-N-MC cells. Biochim Biophys Acta.

[B34] Takahashi M, Fukutake M, Isoi T, Fukuda K, Sato H, Yazawa K, Sugimura T, Wakabayashi K (1997). Suppression of azoxymethane-induced rat colon carcinoma development by a fish oil component, docosahexaenoic acid (DHA). Carcinogenesis.

[B35] Kafrawy O, Zerouga M, Stillwell W, Jenski LJ (1998). Docosahexaenoic acid in phosphatidylcholine mediates cytotoxicity more effectively than other omega-3 and omega-6 fatty acids. Cancer Lett.

[B36] Bradley MO, Swindell CS, Anthony FH, Witman PA, Devanesan P, Webb NL, Baker SD, Wolff AC, Donehower RC (2001). Tumor targeting by conjugation of DHA to paclitaxel. J Control Release.

[B37] Zerouga M, Stillwell W, Jenski LJ (2002). Synthesis of a novel phosphatidylcholine conjugated to docosahexaenoic acid and methotrexate that inhibits cell proliferation. Anticancer Drugs.

[B38] Connolly JM, Gilhooly EM, Rose DP (1999). Effects of reduced dietary linoleic acid intake, alone or combined with an algal source of docosahexaenoic acid, on MDA-MB-231 breast cancer cell growth and apoptosis in nude mice. Nutr Cancer.

[B39] Bogenrieder T, Herlyn M (2003). Axis of evil: molecular mechanisms of cancer metastasis. Oncogene.

[B40] Stennicke HR, Salvesen GS (1998). Properties of the caspases. Biochim Biophys Acta.

[B41] Williams EE, Jenski LJ, Stillwell W (1998). Docosahexaenoic acid (DHA) alters the structure and composition of membranous vesicles exfoliated from the surface of a murine leukemia cell line. Biochim Biophys Acta.

[B42] Scherer JM, Stillwell W, Jenski LJ (1997). Spleen cell survival and proliferation are differentially altered by docosahexaenoic acid. Cell Immunol.

[B43] Zerouga M, Stillwell W, Stone J, Powner A, Jenski LJ (1996). Phospholipid class as a determinant in docosahexaenoic acid's effect on tumor cell viability. Anticancer Res.

[B44] Dratz EA, Deese AJ, Simopoulos AP, Kifer RR, Martin RE (1986). The role of docosahexaenoic acid (22:6ω3) in biological membranes: examples from photoreceptors and model membrane bilayers. Health Effects of Polyunsaturated Fatty Acids in Seafoods.

[B45] Salem NJ, Kim HY, Yergey JA, Simopoulos AP, Kifer RR, Martin RE (1986). Docosahexaenoic acid: membrane function and metabolism. Health Effects of Polyunsaturated Fatty Acids in Seafoods.

[B46] Balasubramanian SV, Campbell RB, Straubinger RM (2002). Propofol, a general anesthetic, promotes the formation of fluid phase domains in model membranes. Chem Phys Lipids.

[B47] Stillwell W, Wassall SR (2003). Docosahexaenoic acid: membrane properties of a unique fatty acid. Chem Phys Lipids.

[B48] Siddiqui RA, Jenski LJ, Neff K, Harvey K, Kovacs R, Stillwell W (2001). Docosahexanoic acid induces apoptosis in Jurkat cells by a protein phosphatase-mediated process. Biochim Biophys Acta.

[B49] Siddiqui RA, Jenski LJ, Wiesehan JD, Hunter MV, Kovacs RJ, Stillwell W (2001). Prevention of docosahexaenoic acid-induced cytotoxicity by phosphatidic acid in Jurkat leukemic cells: the role of protein phosphatase-1. Biochim Biophys Acta.

