# Supplementary figures and images for: Anticancer properties of propofol-docosahexaenoate and propofol-eicosapentaenoate on breast cancer cells
Source: Breast Cancer Res. 2005 Jun 7;7(5):R645–54. doi: 10.1186/bcr1036 (PMC1242121; doi:10.1186/bcr1036)

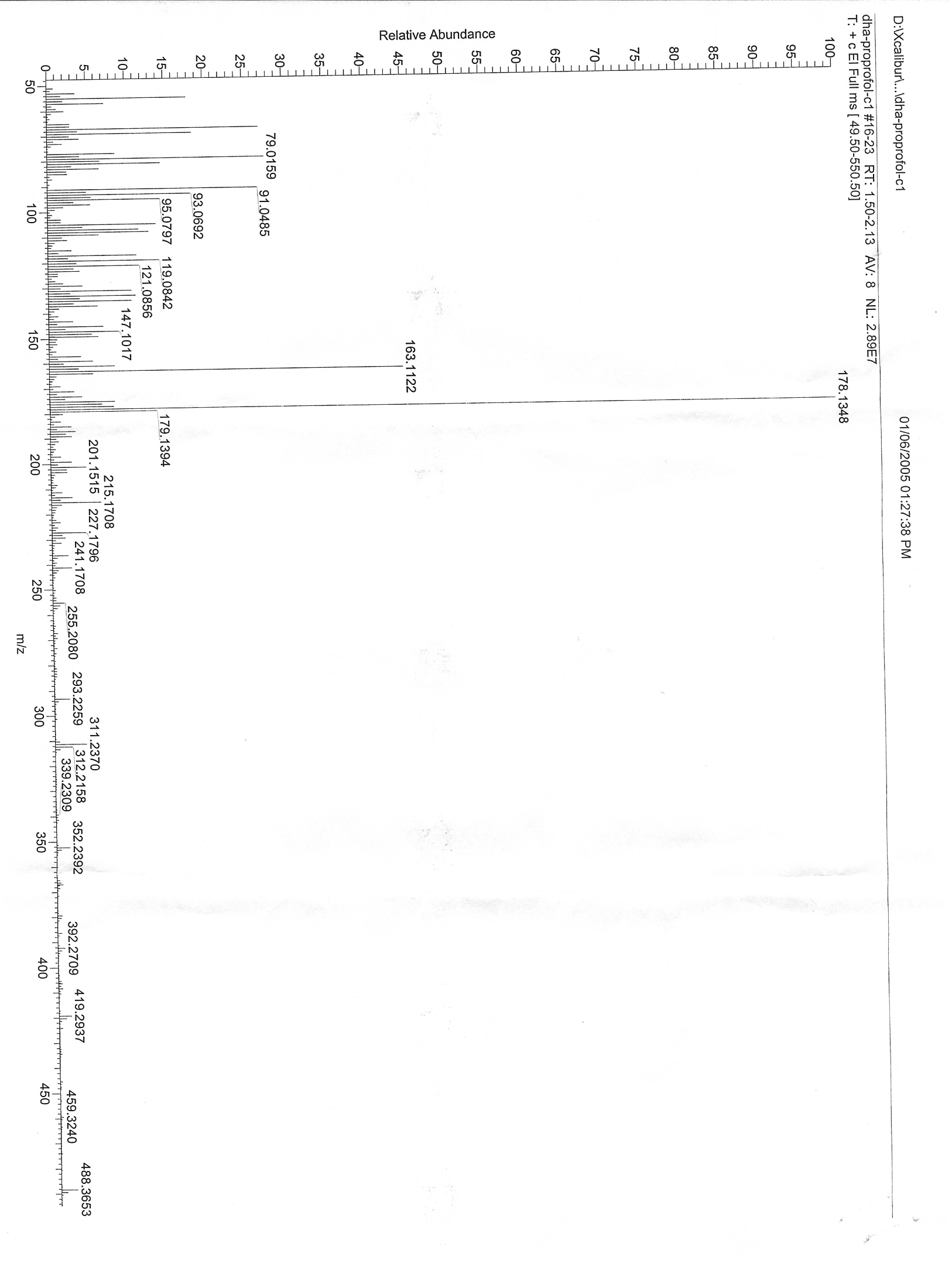


Propofol-DHA


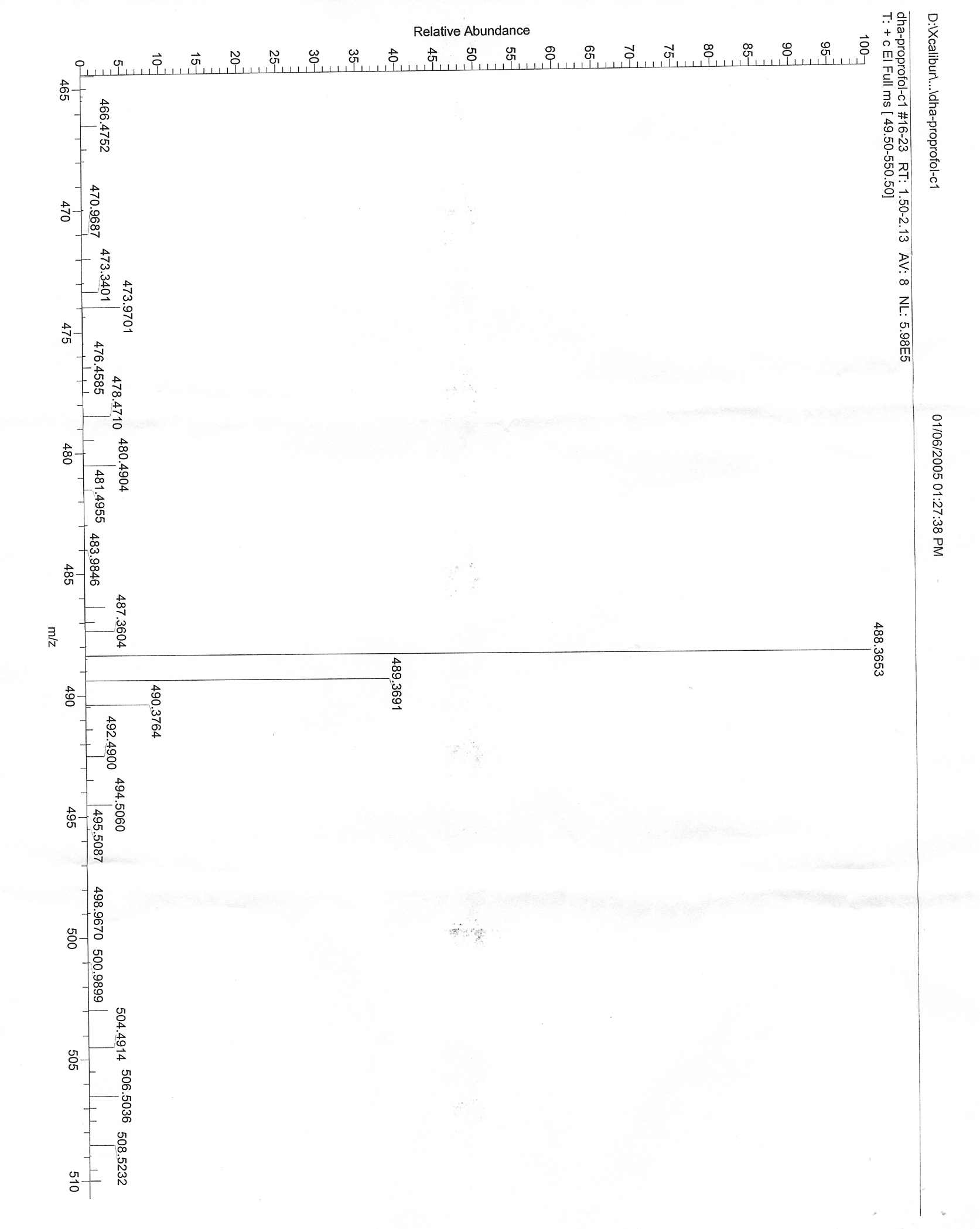


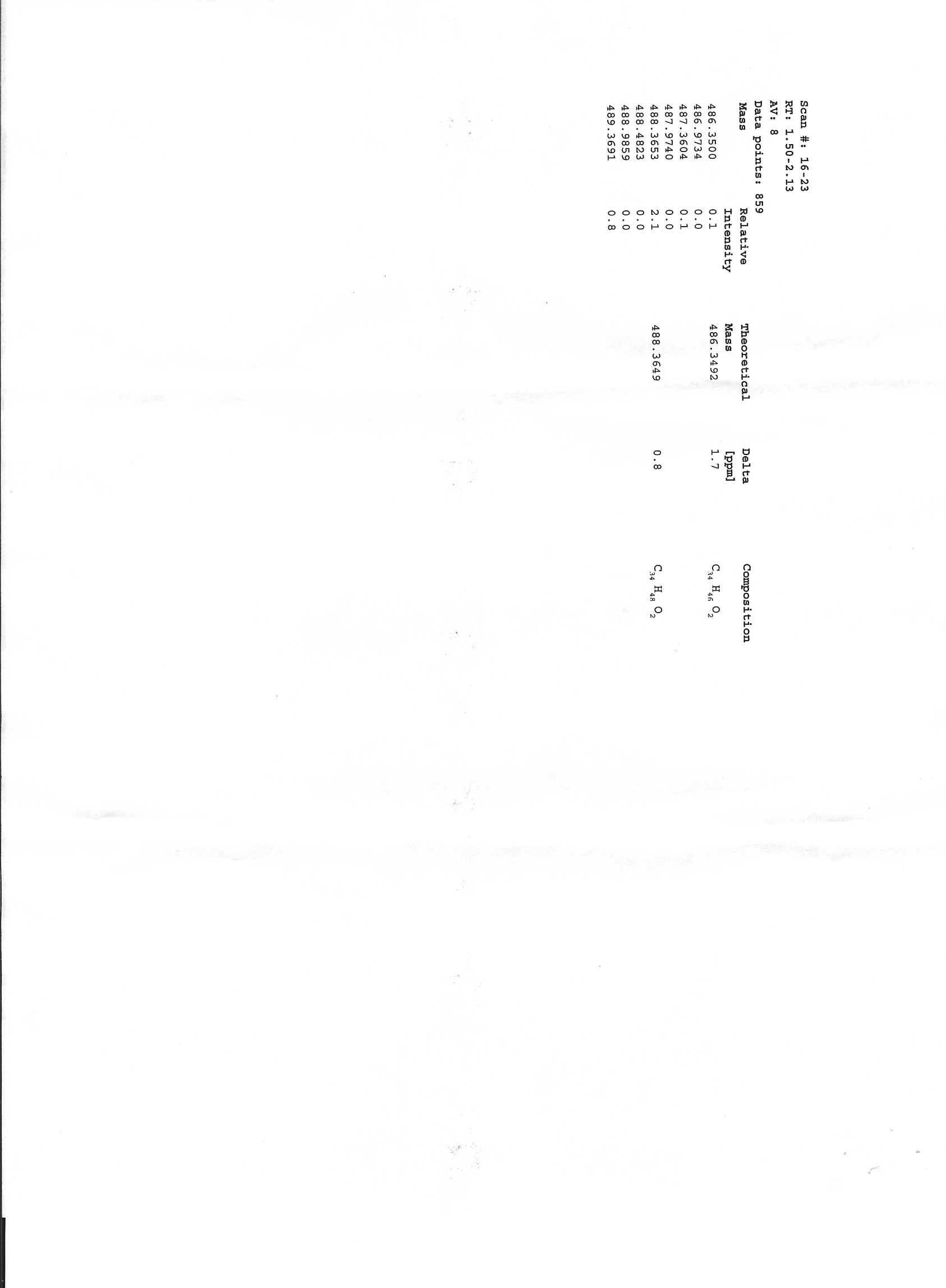


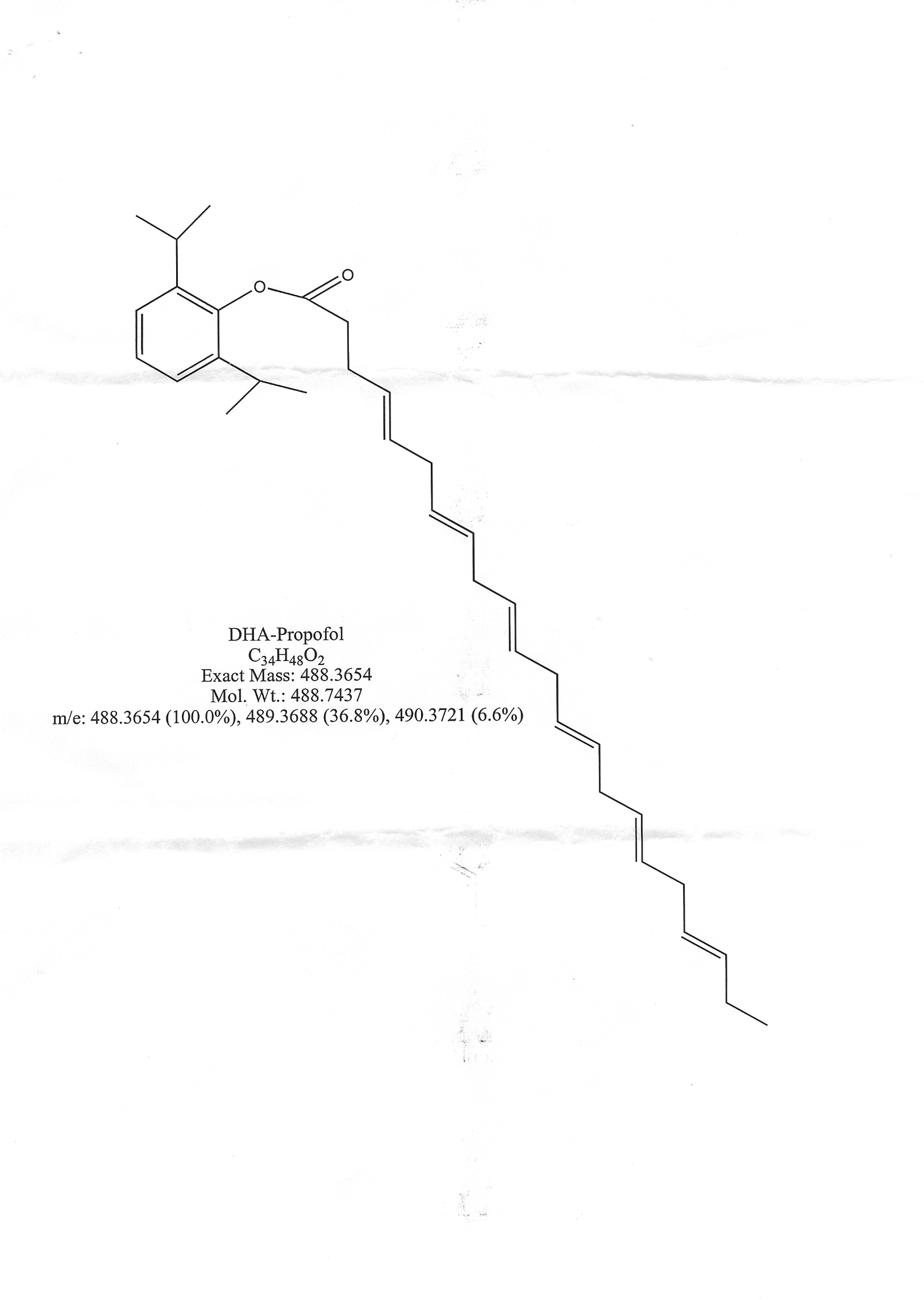


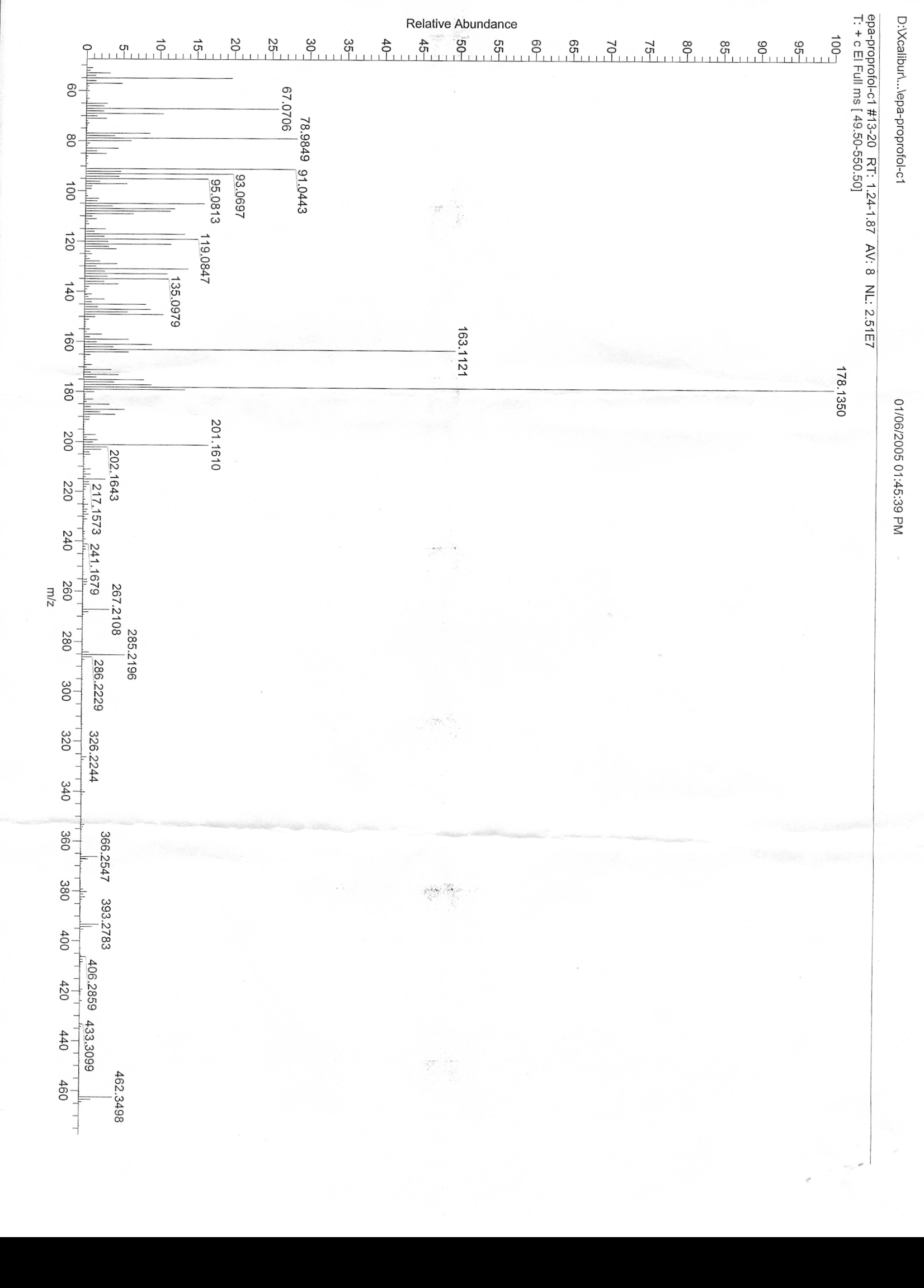


Propofol-EPA


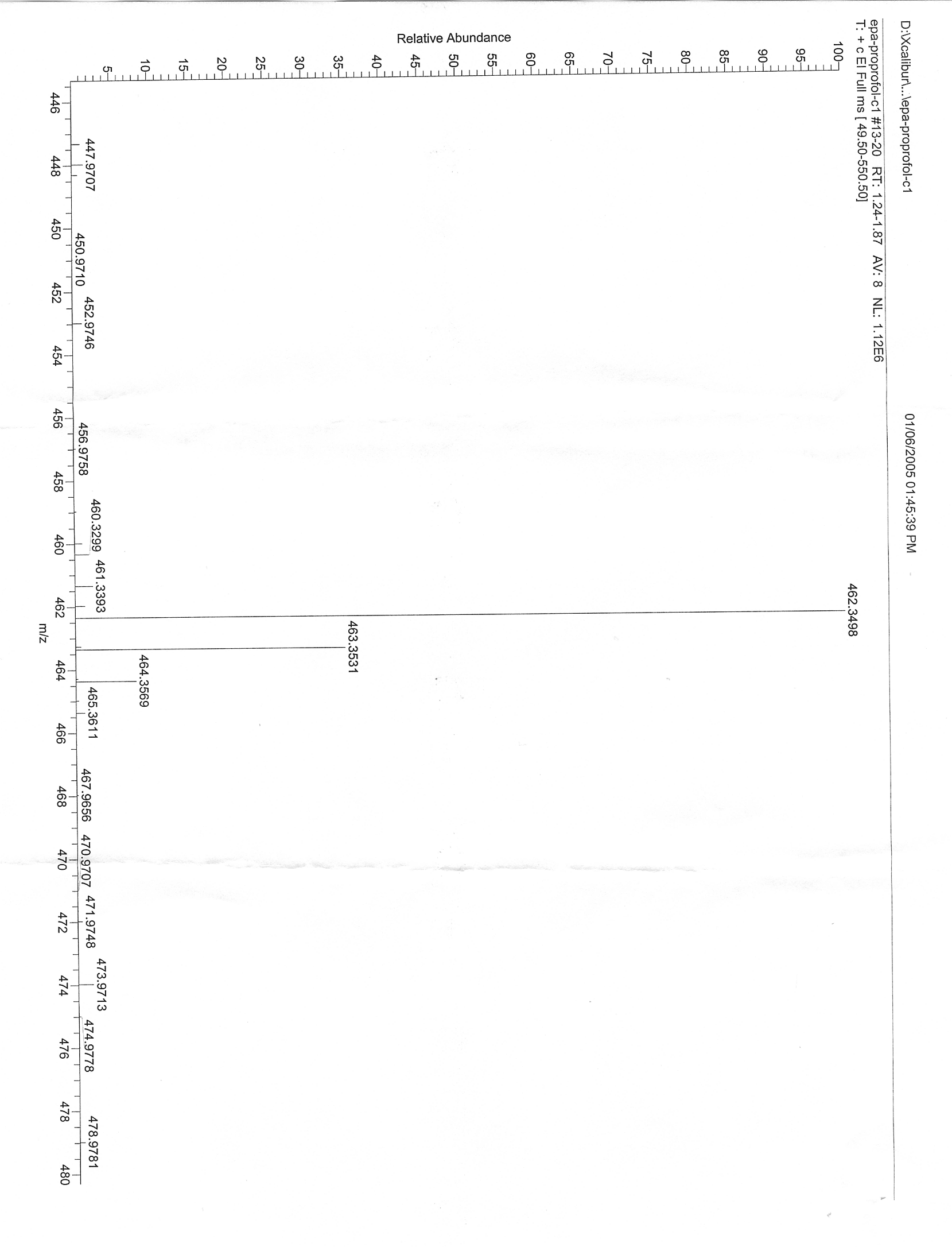


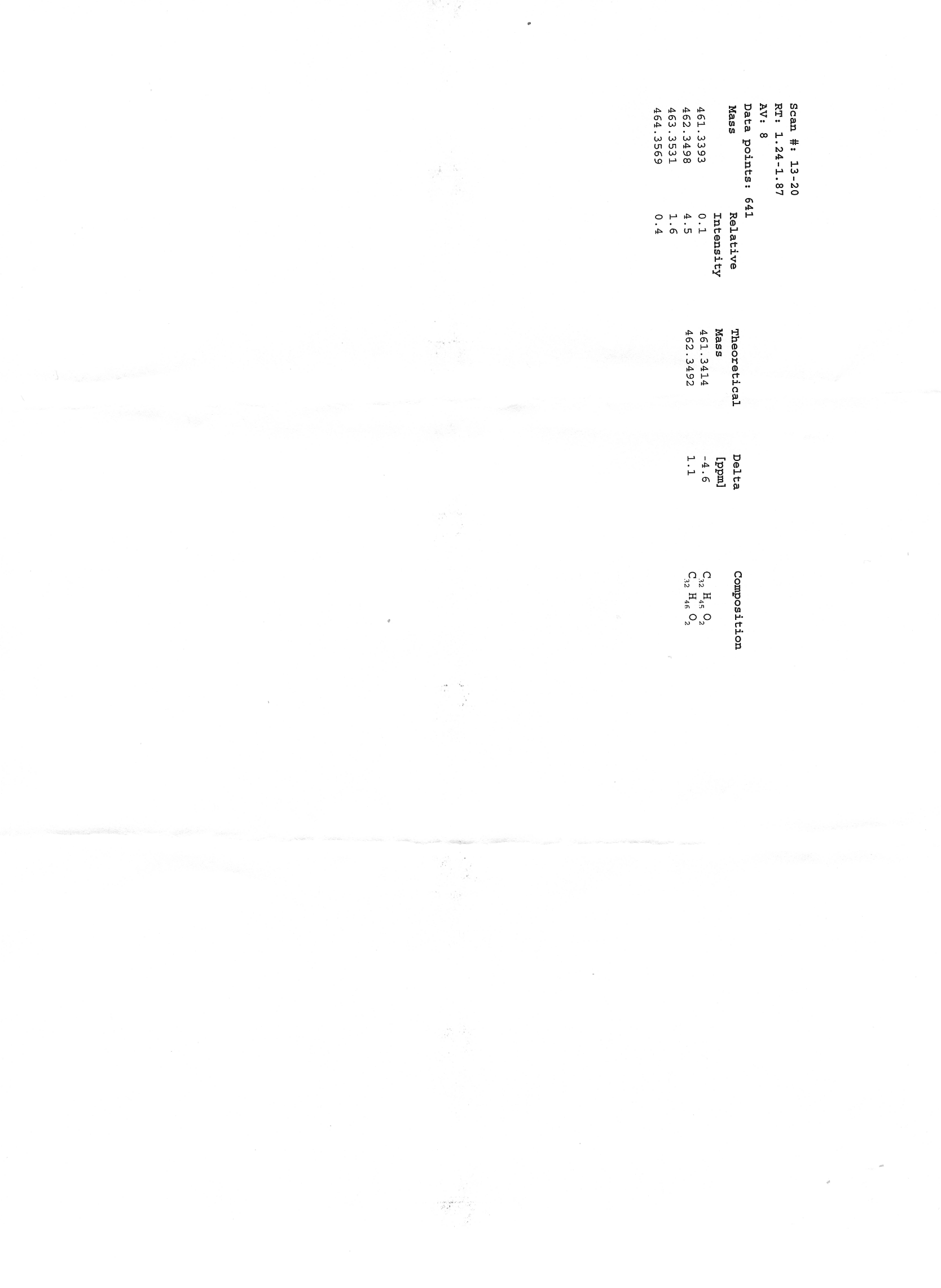


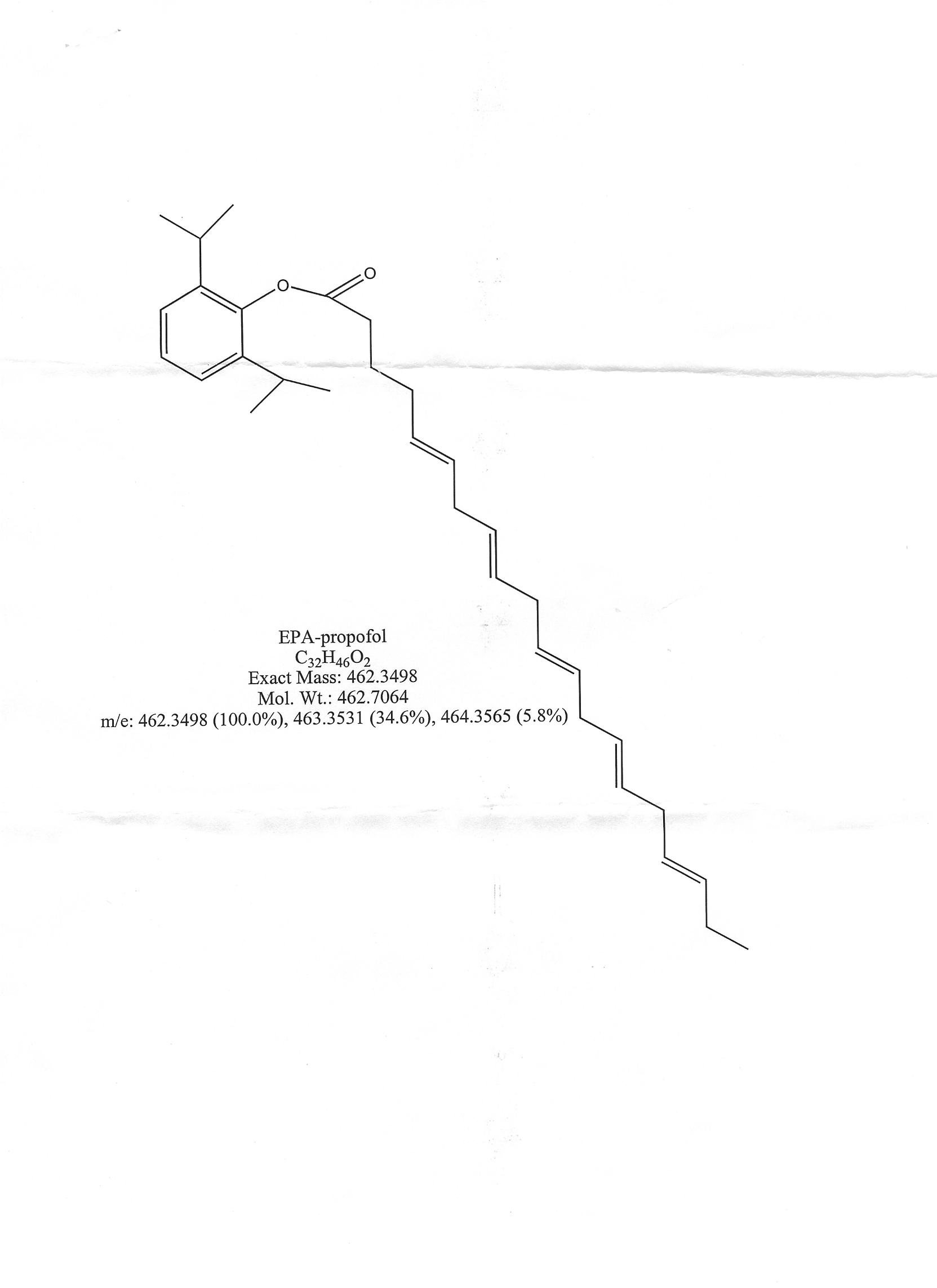

Supplement: Additional File 1 — A Word document containing mass spectrometry data for propofol-DHA and propofol-EPA conjugates are presented as additional file 1. [file bcr1036-S1.doc]
